# Passive Internet of Events Enabled by Broadly Compatible Self‐Powered Visualized Platform Toward Real‐Time Surveillance

**DOI:** 10.1002/advs.202304352

**Published:** 2023-10-23

**Authors:** Chaojie Chen, Haoran Zhang, Guoqiang Xu, Tingting Hou, Jingjing Fu, Haoyu Wang, Xin Xia, Cheng Yang, Yunlong Zi

**Affiliations:** ^1^ Department of Mechanical and Automation Engineering The Chinese University of Hong Kong Shatin N.T. Hong Kong Hong Kong China; ^2^ Thrust of Sustainable Energy and Environment The Hong Kong University of Science and Technology (Guangzhou) Nansha Guangzhou Guangdong 511400 China; ^3^ Institute of Materials Research Tsinghua Shenzhen International Graduate School Tsinghua University Shenzhen 518055 P. R. China; ^4^ HKUST Shenzhen‐Hong Kong Collaborative Innovation Research Institute Futian Shenzhen Guangdong 518048 China; ^5^ Guangzhou HKUST Fok Ying Tung Research Institute Guangzhou Guangdong 511400 China

**Keywords:** passive IoE, self‐powered visualized platform, surveillance, triboelectric nanogenerators, wireless sensing

## Abstract

Surveillance is an intricate challenge worldwide especially in those complicated environments such as nuclear plants, banks, crowded areas, barns, etc. Deploying self‐powered wireless sensor nodes can increase the system's event detection capabilities by collecting environmental changes, while the incompatibility among components (energy harvesters, sensors, and wireless modules) limits their application. Here, a broadly compatible self‐powered visualized platform (SPVP) is reported to construct a passive internet of events (IoE) network for surveillance systems. By encoding electric signals into reference and working LEDs, SPVP can visualize resistance change generated by commercial resistive sensors with a broad working range (<10^7^ Ω) and the transmission distance is up to 30 meters. Visible light signals are captured by surveillance cameras and processed by the cloud to achieve real‐time event monitoring and identification, which forms the passive IoE network. It is demonstrated that the passive‐IoE‐based surveillance system can detect intrusion, theft, fire alarm, and distress signals quickly (30 ms) for 10^6^ cycles. Moreover, the confidential information can be encrypted by SPVPs and accessed through a phone application. This universal scheme may have huge potential for the construction of safe and smart cities.

## Graphical abstract

A broadly compatible self‐powered visualized platform is designed to construct passive internet of events network for high security surveillance system. This system can detect events, such as human intrusion, theft, fire alarm, and rescue in real time. Simultaneously, information can be encoded and transmitted over 30 meters in the network.

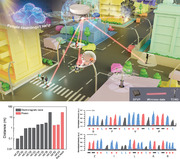

## 1 Introduction

Intelligent security systems are essential equipment for preventing violence, attacks, crimes, theft, vandalism, terrorism, etc.^[^ ^1^ ^]^ Among them, video surveillance system is a popular solution that employs distributed cameras to monitor events happening at a certain place in real time.^[^ ^2^ ^]^ In this system, event detection is essential to identify the potential threat in a place. Conventionally, it requires people to monitor the live stream continuously and recognize/classify the happening events, which is time‐consuming and inefficient.^[^ ^3^ ^]^ To overcome the above challenges, artificial intelligence technology was introduced to recognize human faces and motion, which can efficiently detect and record ongoing events or individuals with less manpower.^[^ ^4^ ^]^ However, the overlap between objects, diverse human behavior, and especially the uncovered area of cameras cannot provide sufficient information, making it challenging to analyze and detect events. Deploying more cameras may address this issue but may not be feasible, considering the increasing cost in installation and maintenance. Simultaneously, it would lead to “surveillance fatigue” among security department, where the sheer volume of footage reduces the monitoring and analyzing efficiencies.

Sensors can be massively deployed to compensate the deficiency of cameras, and thus can critically promote the surveillance efficiency by providing physical or chemical changes induced by the events.^[^ ^5^ ^]^ As the number of sensors increases, recharging and maintaining billions of batteries become infeasible, and the influence of hazardous components in battery materials on the environment cannot be ignored.^[^ ^6^ ^]^ The passive internet of things (IoT) may address this issue using wireless battery‐free distributed devices, without the limitation from batteries.^[^ ^7^ ^]^ Energy harvesting technologies play an irreplaceable role in passive IoT as they can harvest solar, thermal, and mechanical energy to make devices self‐powered and form the passive network. Among these energy harvesters, triboelectric nanogenerators (TENGs) attract much attention due to advantages of low‐cost, high output and flexible.^[^ ^8^ ^]^ They can harvest energy from multiple mechanical energy sources (human motion, wind, vibrations, etc.) and enable self‐powered wireless systems (SPWSs).^[^ ^9^ ^]^

Previous studies demonstrated that signals can be transmitted between TENG‐based SPWSs and receiver circuits through electromagnetic induction^[^ ^10^ ^]^ and photons.^[^ ^11^ ^]^ However, the incompatibility of TENGs with commercial sensors and wireless transmission modules hinders the development of SPWSs. Firstly, the pulsed current generated by TENGs makes the system frequency‐dependent and deteriorates the real‐time sensing ability of sensors. Simultaneously, the higher impedance of TENGs cannot match well with low‐resistance commercial sensors, which restricts the available types of sensors.^[^ ^12^ ^]^ Power management modules and chips that provide a continuous and stable power supply can address the above issues, but the introduction of them increases the complexity and power consumption of the system. Thirdly, sensing signals are usually transmitted through wireless modules like Bluetooth and WiFi, which needs much additional power.^[^ ^13^ ^]^ Systems need to be charged for a long time to transmit signals once, which is time‐consuming and inefficient.^[^ ^14^ ^]^ Therefore, developing a wireless platform that is broadly compatible with all components well for real‐time event detection is challenging.

**Figure 1:**
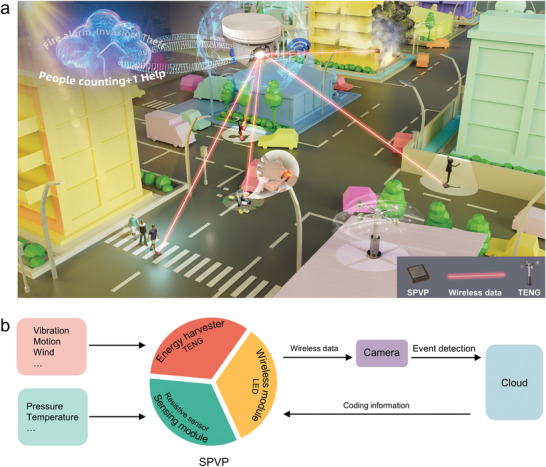
Broadly compatible self‐powered visualized platform. a) Passive internet of events that monitor people, thief, injured person, and fire. The event information is monitored by sensor modules and transmitted to cameras by LEDs when TENGs are mechanically triggered. b) Block diagram of the system. The energy harvesters (TENGs) can convert environmental mechanical energy to electricity that supports the operation of resistive sensors. The LED module can wirelessly transmit resistive measurements of sensors to cameras. The cloud with developed software supports real‐time event detection and display, data storage, and information coding.

**Figure 2:**
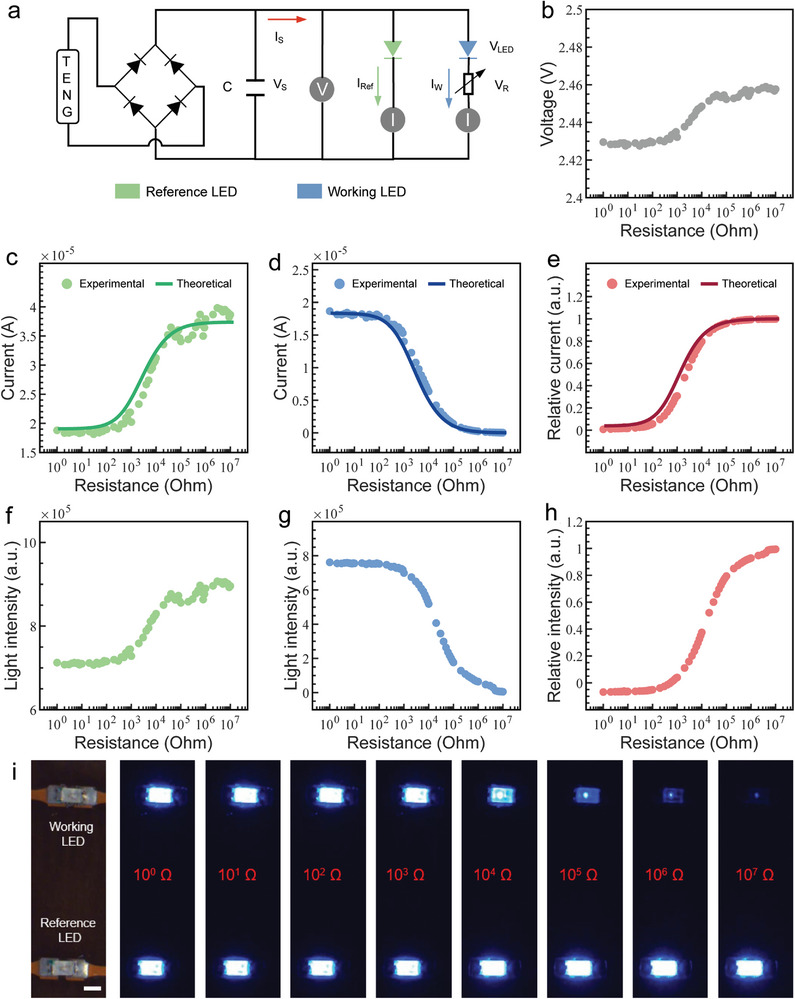
The electrical and optical behaviors of the SPVP. a) Schematic of the equivalent circuit. b) Output voltage. Experimental and theoretical currents of c) the reference LED and d) working LED. e) Experimental and theoretical relative current. Total light intensity of f) the reference LED and g) working LED. h) Relative intensity. i) Photographic images of the reference LED and working LED, and their light intensity at different load resistances. Scale bar, 1 mm.

Herein, we report a highly compatible self‐powered visualized platform (SPVP) to establish passive internet of event (IoE) networks for high‐security surveillance systems (**Figure** **1**a). The SPVP is fabricated by integrating a rich variety of TENGs, commercial resistive sensors, and reference/working light emitting diodes (LEDs), achieving electro‐optical conversion of sensing signals for resistive sensors (Figure 1b). An electric module is designed to not only address challenges in power fluctuations of TENG, but also transform electric signals into optical domain by comparing light intensities in reference and working LEDs (**Figure** **2**a). We demonstrated that real‐time pressure and temperature sensing signals can be wirelessly transmitted via SPVPs by introducing commercial pressure sensors and thermistors, showing good event identification capability. The maximum transmission distance is up to 30 m. With this, the passive IoE‐based high‐security system can be developed by integrating SPVPs, video surveillance system, and the cloud (Figure 1a). When the deployed sensors are triggered, SPVPs can send event information via emitting visible light, which can be received by the surveillance cameras and processed by the cloud for event recognition. Consequently, the IoE system can efficiently identify multiple events including human intrusion, theft, fire alarm, and rescue in real time. Furthermore, confidential information such as Morse code and website address can be encoded into SPVPs, which can be read through a designed phone application. This conceptual study presents a prototype to construct a passive IoE network using our broadly compatible SPVPs for high‐efficient event identification. It addresses the issues in system integration, model estimation, information encoding and decoding, which is promising to develop a general and simple solution for next‐generation high‐security surveillance systems.

## 2 Results and Discussion

### 2.1 Working Principle of SPVP

The equivalent circuit of SPVP is shown in Figure 2a. It comprises of the rotation TENG (R‐TENG), a rectifier, a capacitor, a reference LED, resistive sensor, and a working LED. The triboelectrification between polytetrafluoroethylene (PTFE) and polyethylene terephthalate (PET) produces charge transfer of 400 nC and the short‐circuit current of R‐TENG is 50 µA (Figure S1, Supporting Information). Moreover, the R‐TENG can work more than 1.1 × 10^6^ cycles without attenuation (Figure S2, Supporting Information), which is able to support long time operation of the SPVP. Considering its frequency‐dependent property may deteriorate the real‐time sensing performance, a capacitor, which has a fast charge/discharge capability and long cycle life,^[^ ^15^ ^]^ is used to store energy and stabilize the electrical and optical outputs of LEDs. As a result, the circuit with a capacitor can maintain the output voltage and current at certain values, giving a flicker‐free light (Figure S3 and Movie S1, Supporting Information). To accurately transplant the function of resistive sensors, the optimized working conditions should be satisfied. Basically, two methods were used frequently to describe the signal change of resistive sensors, including the current measurement with a constant voltage or the voltage measurement with a constant current. Here, a reference LED was used to regulate the output voltage around the turn‐on voltage of LED, with limited variations. For instance, the blue LED can regulate the voltage to be always ≈2.43–2.46 V as the load resistance increases (Figure 2b), and the red and green LEDs regulate the output voltage at 1.67 V and 2.15 V (Figure S4, Supporting Information), respectively. In contrast, the circuit without a reference LED experiences a charge process when the load resistance increases or a discharge process when the load resistance becomes zero (see Figure S5, Supporting Information). The long‐time charge process and instantaneous discharge may damage the LEDs, resulting in poor reliability and long recover time. Therefore, the reference LED can provide a relatively stable working condition for resistive sensors. Next, a working LED is in series with sensors, which are in parallel with the reference LED. It is noted that the light intensity of the LED is proportional to the driving frequency and thus has an input‐dependent behavior (Figure S6, Supporting Information). To eliminate the interference from TENG output, we introduced the relative light intensity between the reference LED and working LED for information transmission. This is because the relative light intensity is solely determined by the relative current change between two branches when the resistance of sensors is changing. Therefore, our design satisfies the working condition of commercial resistive sensors and provides a reliable index for electro‐optical conversion.

To quantitatively analyze the SPVP, the electrical and optical signals at different resistances were measured. The current of the reference LED (I_Ref_) increases as the load resistance increases (Figure 2c), while that of the working LED (I_W_) decreases when the resistance increases (Figure 2d), which is caused by the shunt effect in parallel. The relative current change ΔI = between the reference LED and working LED is calculated and shown in Figure 2e, which is proportional to the load resistance. Relevant time‐dependent signals are also presented (Figure S7, Supporting Information). Correspondingly, in Figure 2f, the total light intensity of the reference LED (TLI_Ref_) increases as the resistance increases. In contrast, the total light intensity of the working LED (TLI_W_) decreases as the resistance increases (Figure 2g). The relative light intensity (ΔTLI) between the reference LED and the working LED also shows a proportional relationship with the resistance (Figure 2h). Therefore, the behaviors of optical signals are consistent with the current changes. Moreover, the theoretical results of the equivalent circuit analysis are fitted well with the experimental results, providing a design principle for SPVP (Note S1, Supporting Information). In Figure 2i, the photographic images of the reference LED and working LED at different load resistances are recorded, showing the pronounced light intensity change. It should be noted that our design enables the SPVP to have a wide working range of the resistance from 10^0^ to 10^7^ Ω, which is all smaller than the output impedance of R‐TENG (200 MΩ in Figure S8, Supporting Information), indicating that the operation of SPVP is not affected by the interference of TENG's impedance. Importantly, this wide working range is compatible with most resistive sensors in general and thus shows good extensibility of this system.^[^ ^16^ ^]^

### 2.2 Integrating Commercial Sensors into SPVP

**Figure 3:**
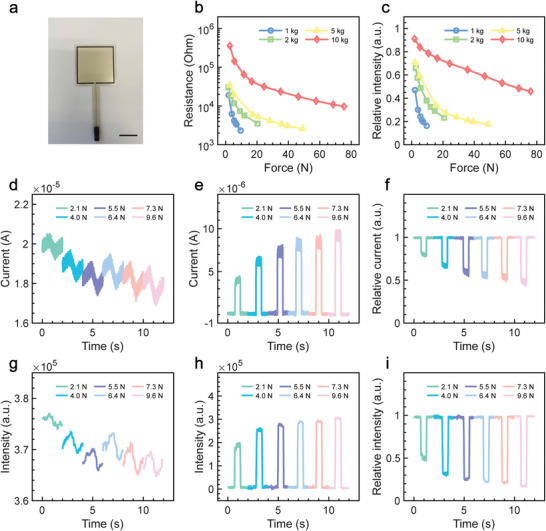
Integrating pressure sensors with SPVP. a) Photographic image of commercial pressure sensor. Scale bar, 20 mm. The b) resistance and c) relative intensity of sensors at different loading forces. Current signals of d) the reference LED and e) the working LED. f) Relative current at different loading forces. The total light intensity of g) the reference LED and h) the working LED at different loading force. i) Relative intensity. Signals of d–i) are generated by the sensor featured with a 1 kg working range.

To demonstrate the extensibility of the system, commercial flexible force sensors with different working ranges (1, 2, 5, and 10 kg) were introduced to the SPVP (**Figure** **3**a). Their resistances decrease with the increasing forces whose values are distributed in the range of 10^3^–10^6^ Ω (Figure 3b). Thus, force signals are successfully encoded to the optical domain (Figure 3c; Figures S9–S12, Supporting Information). Current signals and light intensity changes of the reference LED share the same trend that decreases as the force increases (Figure 3d,g). Similarly, current signals and light intensity changes of the working LED have the same trend, which increases when the force increases (Figure 3e,h). It is noted that the response time and recovery time of the SPVP are ≈15 ms, showing a fast response (Figure S13, Supporting Information). Furthermore, the optical response time of the SPVP is 30 ms (Figure S14, Supporting Information), which is longer than the electrical response time. This difference is attributed to the low video sampling rate of 30 fps. The relative electric current (Figure 3f) and relative light intensity (Figure 3i) share the same trend that they both decrease as the force increases. Overall, the electric signal changes are completely mapped in the optical domain, which verifies the electro‐optic conversion capability of SPVPs. Therefore, commercial pressure sensors can be fully transplanted to the SPVP, where it can convert pressure information to distinguishable visible light for wireless signal transmissions. Furthermore, an in situ heating test was conducted to measure the optical signal generated by thermistors with different initial resistances at room temperature (Figure S15a, Supporting Information). Results show that the SPVP embedded with four types of thermistors have distinctive optical response to temperature change in the range from 23 °C to 65 °C (Figure S15b,c and S16–S19, Supporting Information). Overall, the SPVP shows good compatibility with commercial resistive sensors that can detect various physical quantities, enabling good event‐detection ability.

### 2.3 SPVP‐Based Passive IoE Networks

**Figure 4:**
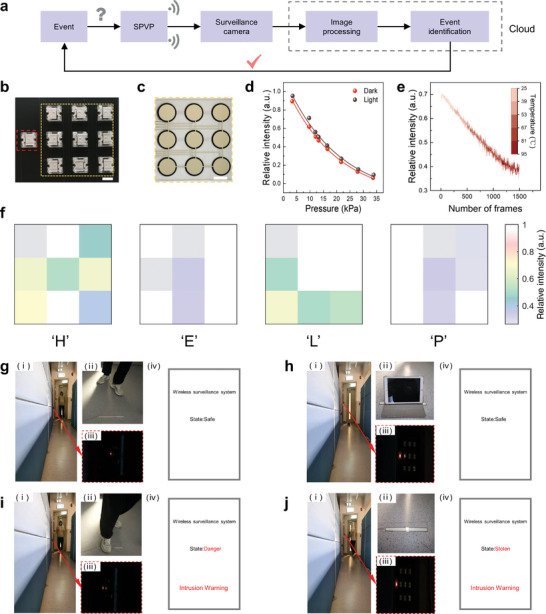
SPVP‐based IoE network for surveillance system. a) A flow chart schematic of the IoE. b) Reference LED (read square) and working LED array (yellow square) of the SPVP. Scale bar, 5 mm. c) Pressure sensor array of the SPVP. Scale bar, 10 mm. d) The relative intensity between the working LED and reference LED under indoor condition and dark condition at different pressures. e) In situ monitoring the environment temperature via a camera. f) The distress signal “HELP” sent by a worker through the SPVP and then decoded by the camera. Real‐time intrusion monitoring: g) safe state and i) intrusion warning. Real‐time anti‐theft monitoring: h) safe state and j) stolen warning.

The IoE‐based security system was developed by integrating SPVPs, surveillance cameras, and cloud. **Figure** **4**a shows the flow chart of the working mechanism of the IoE network, where the unknown event first triggers the sensor of the SPVP, then the electric signals are converted into visible light. Next, cameras capture the optical information and send it to the cloud for the image processing process. Finally, the cloud can identify the input event and give a response. It should be noted that the above working cycle is processed in real time, showing a high efficiency. Here, we prepared a SPVP containing a reference LED, working LED array (Figure 4b) and a pressure sensor array (Figure 4c). As shown in Figure 4d, this SPVP has stable output performance as the relative light intensity of the working LEDs in darkness and indoor light conditions are similar. In fact, the light intensity and pixel number of the dark condition are higher than that of the light condition, showing that the environment light can overwhelm the light intensity of LEDs (Figure S20, Supporting Information). However, the relative light intensities under the two conditions are quite similar, which can be a stable indicator to evaluate the external pressure, indicating the effectiveness of the reference LED that eliminates the environment interference. We also investigated the output performance of R‐TENG with different material combinations and found that the combination of polycarbonate (PC) and fluorinated ethylene propylene (FEP) has the largest output, which is two times larger than the PET and PTFE (Figure S21, Supporting Information). As a result, the PC‐FEP enabled SPVP can work within 2 m under the daylight, while the SPVP cannot work when using PET and PTFE (Figure S22, Supporting Information). This is because the power of LED has been increased (Figure S23, Supporting Information). Therefore, increasing the energy harvesting ability of the system can improve the resistance of the SPVP to the environment.

Interestingly, here we first demonstrated that the IoE network can recognize distress signals, as shown in Figure 4f, the word “HELP” is recognized in real‐time (Movie S2, Supporting Information). This can provide a reliable means of communication in unmanned area or some places like power plant where strong EMI influences the radio communication. Then, a thermistor is introduced to the SPVP that enables the IoE network to monitor the temperature change of the environment for fire alarm. In Figure 4e, we recorded the relative intensity change of the SPVP as the temperature increased from 25 °C to 95 °C, and a real‐time video was recorded (see Movie S3, Supporting Information). To detect the potential intrusion, a pressure sensor is placed on the floor near to the door, then the graphics user interface (GUI) shows that the place is safe when only the reference LED is lighted, which means that no one breaks into the house (Figure 4g). When people walk through the door, the pressure sensor is triggered that one working LED illuminates. Then the GUI indicates that the place is in danger, and an intrusion warning is sent (Figure 4i; Movie S4, Supporting Information). By changing the trigger condition, our IoE network is enabled with anti‐theft function. As shown in Figure 4h, a device is put on the pressure sensor and thus two LEDs are lighted including a reference LED and a working LED, indicating the asset is safe. When taking the device away, the working LED is no longer lighted, which a stolen warning is displayed in the GUI (Movie S5, Supporting Information). Overall, by giving a specific signature and configuration to an SPVP in the cloud, our IoE system can detect relevant events in real time. This hardware trigger mode enables the surveillance system to identify multiple events directly and efficiently.

**Figure 5:**
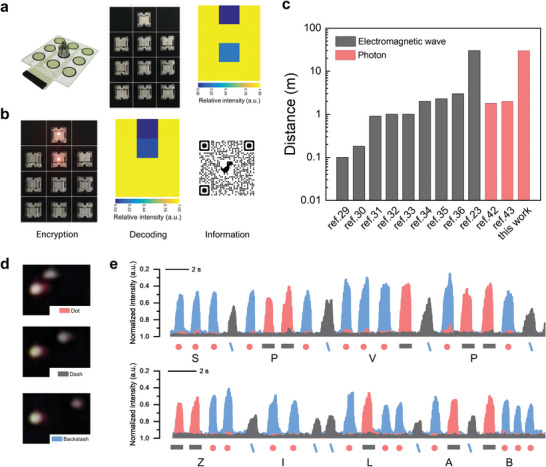
Information encoding and decoding achieved by SPVP‐based IoE network. a) A weight is put on the sensor array and the relative intensity of the SPVP is read by smartphone. b) A website address is encrypted into the SPVP. A smartphone scans the SPVP and reads the encoded information. c) Comparison of the wireless transmission distance of self‐powered system between previous works and our work. Wireless signal types include electromagnetic waves and optical communication. d) Morse codes including ‘.’, ‘–’, and ‘∖’ are realized by three working LEDs with different locations. e) The relative intensity of three working LEDs and corresponding encoded words “SPVP” and “ZI LAB”.

To further demonstrate the feasibility of this IoE, a phone application was designed to identify the optical signals received by the CCD camera, which was sent from the SPVP. In **Figure** **5**a, when putting a weight on the sensor array, the relative light intensity can be read by taking a picture and processed by our phone application (Movie S6, Supporting Information). More importantly, the encryption and transmission of information can be realized by the IoE network in which information is encoded in the cloud and decoded in the mobile terminal. For instance, we first saved a website address in the cloud and set the opening condition for it, such as the website can be opened when the relative intensity is smaller than 0.5. Fixed resistors are used to control the relative light intensity of the SPVP. When the relative light intensity is 0.4 that is smaller than 0.5, we can open the website (Figure 5b), while we cannot open the website when the relative light intensity is 0.7 that is larger than 0.5 (Movie S7, Supporting Information). In this way, we can encode information into the SPVP for encryption and transmission. We show that the real‐time transmission distance of the visible light generated by the SPVP is up to 30 meters, which is achieved by a smartphone with 100X digital zoom. The long transmission distance is comparable to the electromagnetic wave based SPWPs (Figure 5c), but photons are immune to electromagnetic interference which are promising to be employed in factories and power plants where strong EMI occurs. To verify the information transmission capability, the Morse code was converted to the visible light signals which were received by a phone. In Figure 5d, the reference LED keeps lighting and three working LEDs in different locations are used to represent ‘.’, ‘–’, and ‘∖’. The single backslash ‘∖’ and double backslash “∖∖” are used to separate each letters and words respectively. Consequently, this setup successfully transmits words “SPVP” and “ZI LAB” to a smartphone 30 m away and the information can be decoded in real‐time (Figure S24 and Movie S8, Supporting Information). As shown in Figure 5e, the relative intensity of three LEDs is extracted from videos and thus each symbol can be distinguished in the time domain. Importantly, these studies demonstrate the capability of event identification of SPVP‐based IoE network as well as information encryption and transmission for security surveillance.

Traditional wireless sensor nodes can be massively deployed and transmit sensing signals to the cloud wirelessly. But the frequent charging and replacement of batteries over the long lifetime of sensors requires much manpower and cost. Although energy harvesting technology can supply power to those devices, the wireless module cannot transmit electromagnetic waves in real time due to its high‐power consumption. Moreover, the scarcity of band resources and EMI limits the application of radio frequency (RF) based communications especially as the number of distributed devices goes to billions.^[^ ^17^ ^]^ Our state‐of‐the‐art SPVP building blocks can complete the electrical‐optical conversion of sensing signals in real time using low‐power LEDs. The visible light is compatible with the existing closed‐circuit television (CCTV) systems, and we can achieve one‐to‐one, one‐to‐many, and many‐to‐many interactions between cameras and SPVPs without extra infrastructure. Importantly, the visible light communication (VLC) is proved to be high‐speed, low‐cost, and high accuracy.^[^ ^18^ ^]^ Combining the advantages of TENG and VLC technologies, our SPVP‐based IoE network can be a new paradigm that may revolutionize the future of internet of things technology. Although we used the surveillance system as a proof of concept, the IoE network are promising for numerous other applications. For example, the cloud can program SPVPs with location information for people scanning and reading, which can provide precise positioning in a crowded city. Deploying SPVPs at the entrance and exit of the building enable IoE network to count the flow of people. Some biological devices, such as glucose and ion sensors, use i‐t curves for sensing, which is essentially caused by a change in resistance.^[^ ^19^ ^]^ So it is possible to combine them with SPVPs for converting biological signals to optical information. It can be expected that people use phones to scan SPVP‐based medical devices and understand their health conditions conveniently. To realize above applications, further improvement is needed for SPVPs.

As a matter of fact, the transmission distance is limited by the power of TENG and received cameras. This is because the light intensity of LEDs is determined by the TENG output and influenced by the environment light. Only LED with high light intensity can transmit optical signals longer. Cameras with powerful zoom capability can detect the weak light far way, which compensates the drawback of LED light intensity. For instance, in our experiment, we achieved the 30‐meter Morse code transmission using the 100X digital zoom of a smartphone camera. Therefore, in future, it is favorable to develop high‐performance TENGs and design suitable camera modules for further improving our design. Although we demonstrated the feasibility of the SPVPs‐based IoE network using the R‐TENG, other energy harvesting technologies, such as piezoelectric nanogenerator (PENG), electromagnetic generator (EMG), and solar cell can all be introduced to our platform, providing a reliable power supply. For event monitoring, the image processing algorithm including object detection, optical information decoding, and event identification should be optimized to realize fast response and thus improve the security of the IoE system. In despite of SPVP is compatible with resistive sensors in our demonstration, it is important to transplant more kinds of sensors like capacitance sensors to the platform for satisfying different requirements of applications in the future. Our platform is based on the integration of multi‐disciplines, whose breakthrough may promote further development of SPVPs‐based IoE network.

## 3 Conclusion

In summary, we demonstrated a universal, low‐cost scheme to build IoE networks using SPVPs for efficiently detecting events in the surveillance system. A circuit with reference and working LEDs was designed to satisfy the broad working condition of resistive sensors, enabling SPVP to load electric sensing signals to the optical domain in real time for wireless transmission. Electric signals from different commercial pressure and temperature sensors were successfully reflected by relative light intensity between the reference LED and working LEDs. Sensors within the working range 1–10^7^ Ω can be transplanted into the SPVP, showing the universality of SPVPs. When being integrated with the existing surveillance cameras, optical signals from SPVPs can be collected to achieve one‐to‐one or many‐to‐many interactions. In this way, the IoE network can be built by integrating SPVPs, cameras, and cloud. The happening events trigger distributed SPVPs that encode the event information to visible light, which is received by the cameras and further processed by the cloud to recognize the events eventually, with a long transmission distance (30 m). By introducing different sensors and computer vision technology to the IoE network, the IoE‐based surveillance system is demonstrated to detect many events efficiently in real time. Furthermore, people use a smartphone with the developed application to read information from the SPVP that is encrypted by the cloud. Overall, our solution provides a new paradigm for the integration of distributed sensors nodes, TENG, surveillance system, and image processing technology, which is promising in constructing safe and smart cities.

## 4 Experimental Section

### Materials

Commercial resistive pressure sensors with different working range (1, 2, 5, and 10 kg) were purchased from Changzhou Rouxi Electronic Technology Co., Ltd. SMD LEDs, thermistors and Piranha LEDs were purchased from Shenzhen Beike Trading Co., Ltd.

### Fabrication of R‐TENG

Rotor: A circular acrylic sheet (5 mm) with a radius of 150 mm was selected as the substrate. Using laser to cut trenches and through‐holes on the substrate (Figure S25a, Supporting Information). Then, pasting PET films on each trench. Stator: It was fabricated by commercial PCB technology and shape information was shown in Figure S25b (Supporting Information).

### Measurement

The output voltage, current, and charge of R‐TENG were measured by an electrometer (6514, Keithley). For the compression test, using a linear motor (LinMot) to compress piezoresistive sensors and a force senor (DS2‐100N‐XB, Shenzhen Yilice Technology Co., Ltd.) to record the loading force. A CCD camera (HT‐SUA502C‐T, Shenzhen Huateng Vision Technology Co., Ltd.) was used to capture the images of LED and the exposure time was 17 ms. The light intensity of LEDs was processed by MATLAB. For the in situ heating experiment, a hot plate was used to heat thermistors from 22 °C to 65 °C. The temperature of thermistors was measured by a thermocouple (YET‐610L Xinghua Suma Electric Instrument Co., Ltd.). The resistances of pressure sensors and thermistors were measured by a LCR meter (TH2832) from Changzhou Tonghui Electronic Co. Ltd. The maximum distance of optical communication was measured using a smartphone (HUAWEI P50) with 100x digital zoom. The real‐time event detection was conducted by using MATLAB to obtain and process images from two web cameras. The real‐time Morse decoding was achieved by processing the video through MATLAB.

### Total Light Intensity (TLI) Extraction

The LED area was located using the “bwlabel” function in MATLAB R2020b, and then the sum of R/G/B pixel values were calculated, which was the total light intensity. For example, the blue component was extracted when using blue LED. The relative light intensity ΔTLI was defined as ΔTLI = (*TLI_Ref_* − *TLI_W_*)/*TLI_Ref_*. The optical response of SPVP was extracted frame by frame from the recorded videos using MATLAB.

### Statistical Analysis

All shown data were representative for the samples. All experimental data were processed by MATLAB, expressed as mean ± standard deviation. They were also plotted by MATLAB.

## Conflict of Interest

The authors declare no conflict of interest.

## Author Contributions

C.C. and Y.Z. conceived the idea. C.C. designed and carried out the experiments, and analyzed the results. C.C. and Y.Z. discussed the results. H.Z., G.X., T.H., J.F., H.W., and X.X. helped with the experiments. C.C. wrote the paper. C.C., G.X., C.Y., and Y.Z. discussed and edited the manuscript. C.Y. and Y.Z. supervised the project.

## Supporting information

Supporting Information

Supplemental Movie 1

Supplemental Movie 2

Supplemental Movie 3

Supplemental Movie 4

Supplemental Movie 5

Supplemental Movie 6

Supplemental Movie 7

Supplemental Movie 8

## Data Availability

The data that support the findings of this study are available from the corresponding author upon reasonable request.
