# Supplementary material for: Passive Internet of Events Enabled by Broadly Compatible Self‐Powered Visualized Platform Toward Real‐Time Surveillance
Source: Adv Sci (Weinh). 2023 Oct 23;10(34):2304352. doi: 10.1002/advs.202304352 (PMC10700247; doi:10.1002/advs.202304352)
Supplement: Supplementary file 1 — Supporting Information [file ADVS-10-2304352-s003.pdf]

ADVANCED SCIENCE
Open Access

Supporting Information

for *Adv. Sci.*, DOI 10.1002/advs.202304352

Passive Internet of Events Enabled by Broadly Compatible Self-Powered Visualized Platform Toward Real-Time Surveillance

Chaojie Chen, Haoran Zhang, Guoqiang Xu, Tingting Hou, Jingjing Fu, Haoyu Wang, Xin Xia, Cheng Yang and Yunlong Zi**

Supplementary information

Passive Internet of Events enabled by broadly compatible self-powered visualized platform toward real-time surveillance

Chaojie Chen, Haoran Zhang, Guoqiang Xu, Tingting Hou, Jingjing Fu, Haoyu Wang, Xin Xia, Cheng Yang, Yunlong Zi**

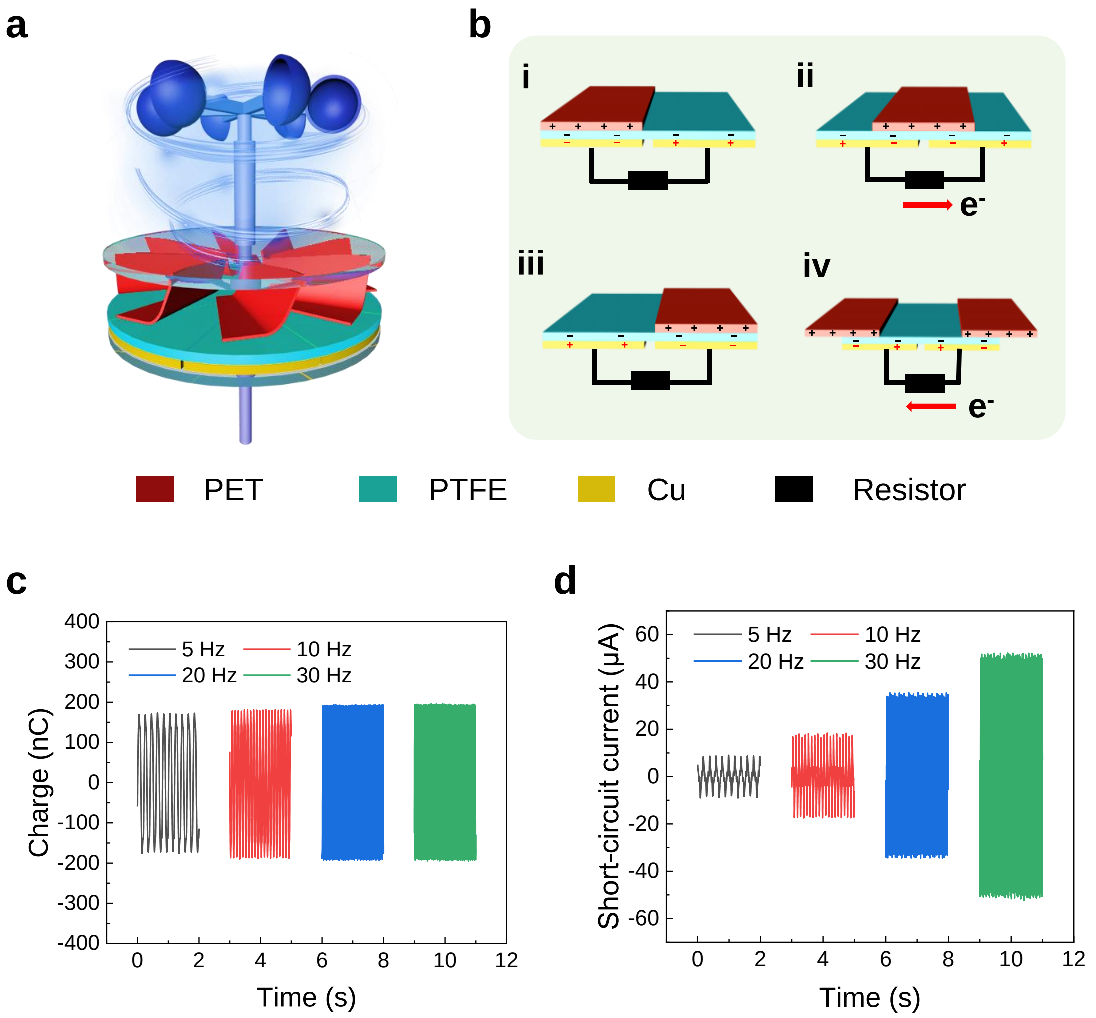

Figure S1. Characterization of the R-TENG. a Schematic diagram. **b** Working principle. **c** Charge and **d** short-circuit current signals.

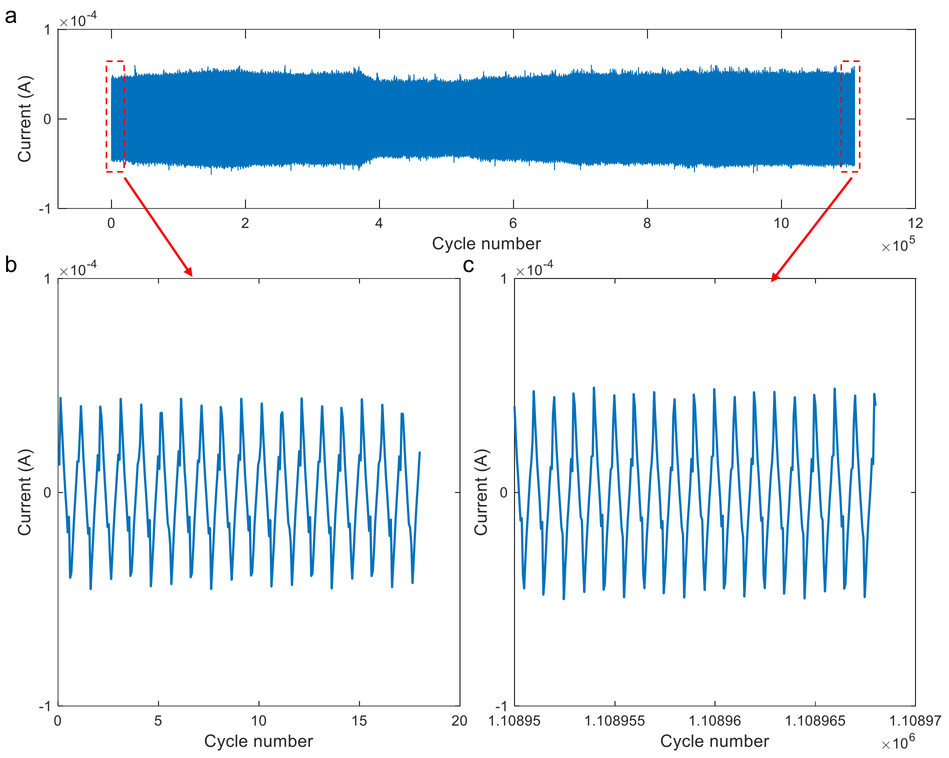

Figure S2 Cycling performance of the R-TENG. a Current output over 1.1×106 cycles. Current curve in the **b** initial stage and **c** end state.

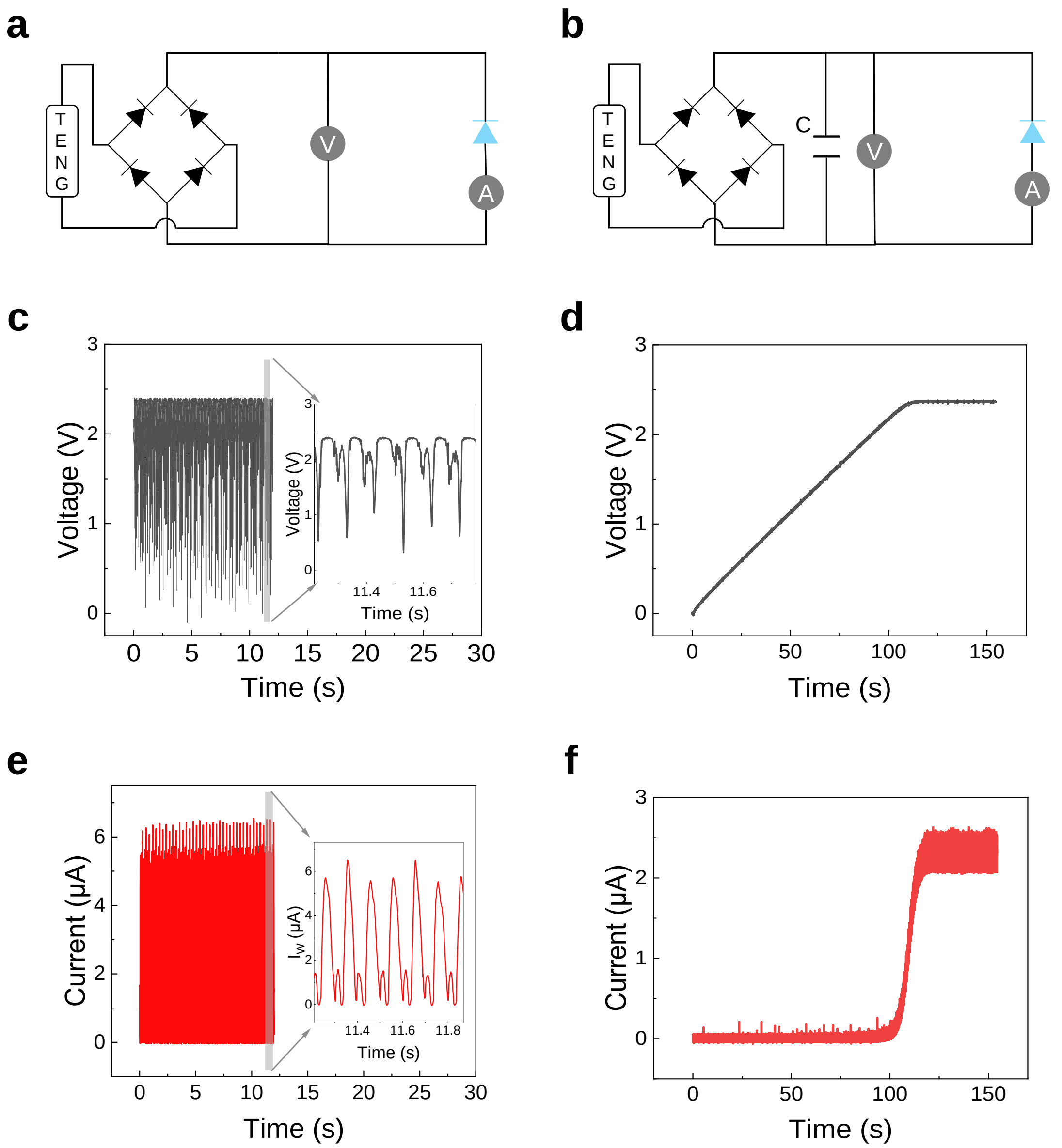

Figure S3. The role of capacitor. Equivalent circuit **a** without and **b** with a capacitor. Frequency-dependent **c** voltage and **e** current signals are obtained by the circuit (a). Stable **d** voltage and **f** current signals are obtained by the circuit (b).

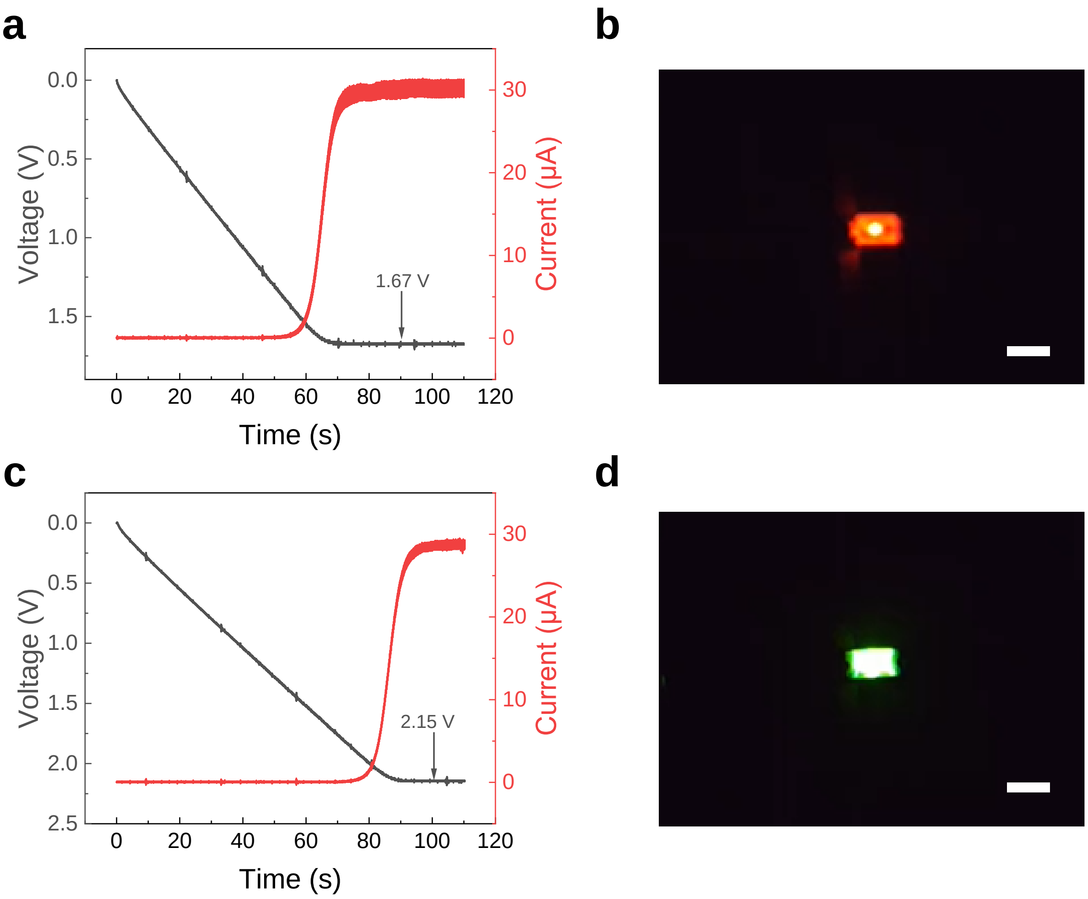

Figure S4. Voltage regulation by different LEDs. a Electrical signals and **b** image of red LED, the stable voltage is ~1.67 V. **c** Electrical signals and **d** image of green LED, the stable voltage is ~2.15 V. Scale bar, 1 mm.

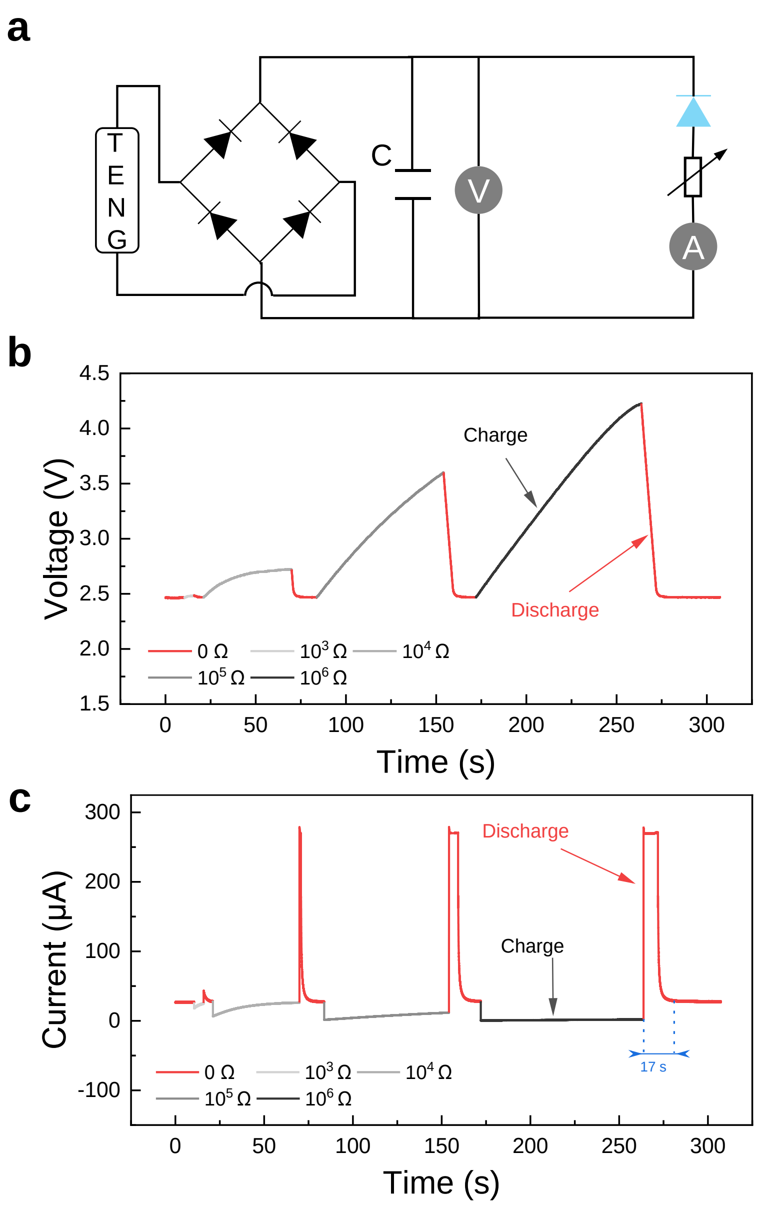

Figure S5. The charge and discharge behavior of the circuit without a reference LED. a Equivalent circuit, **b** voltage signal, and **c** current signal. The capacitor experiences a charging process when the load resistance becomes larger, and it will discharge when the load resistance becomes zero. The relaxation time of capacitor is larger ~17 s after 91 s charging.

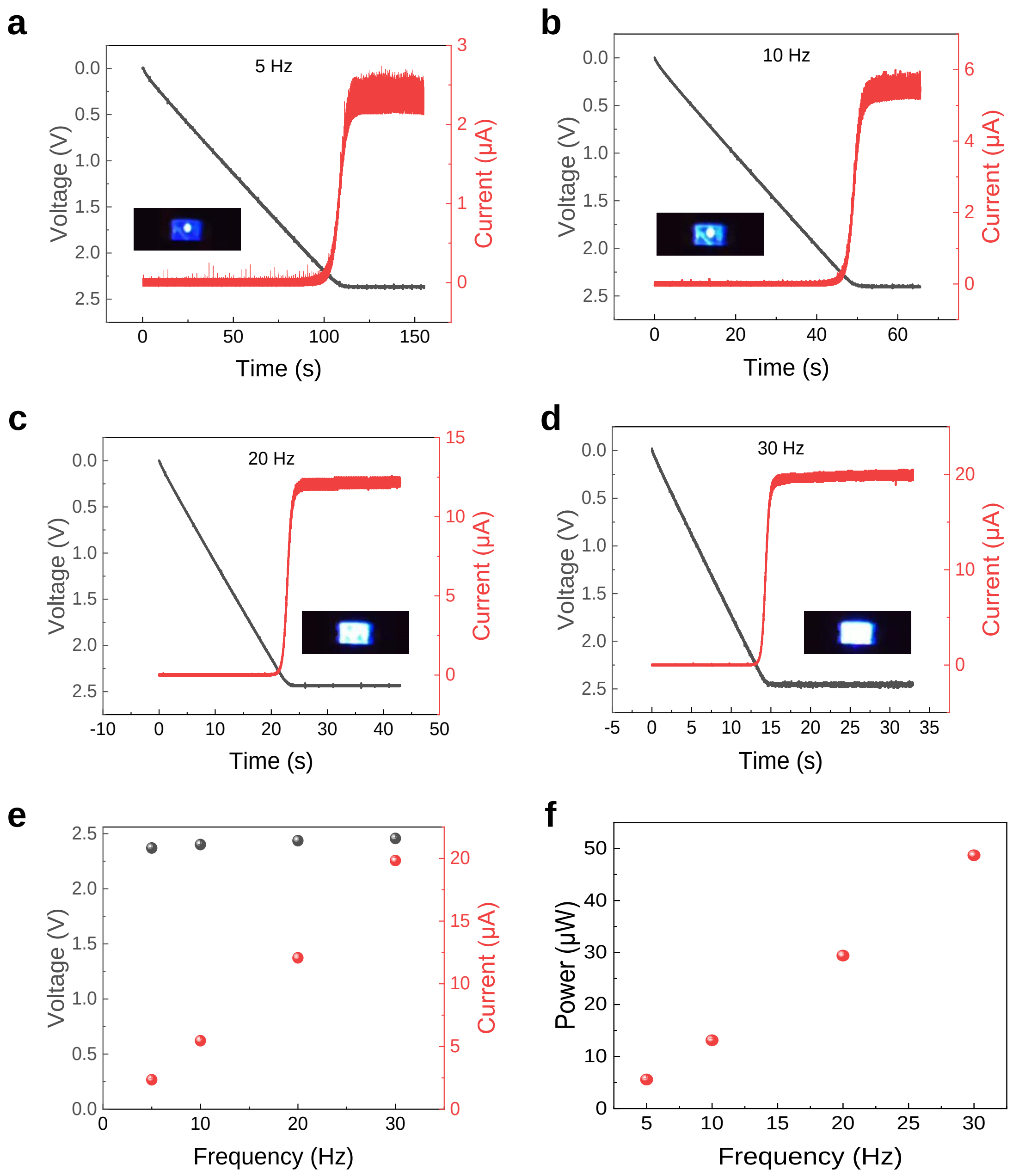

Figure S6. The blue LED is powered by R-TENG at different frequencies: **a** 5 Hz, **b** 10 Hz, **c** 20 Hz, **d** 30 Hz. **e** Summary of voltage and current values at different frequencies. The voltage of blue LED is around 2.45 V regardless of frequency change. **f** The power of LED is proportional to the frequency of R-TENG.

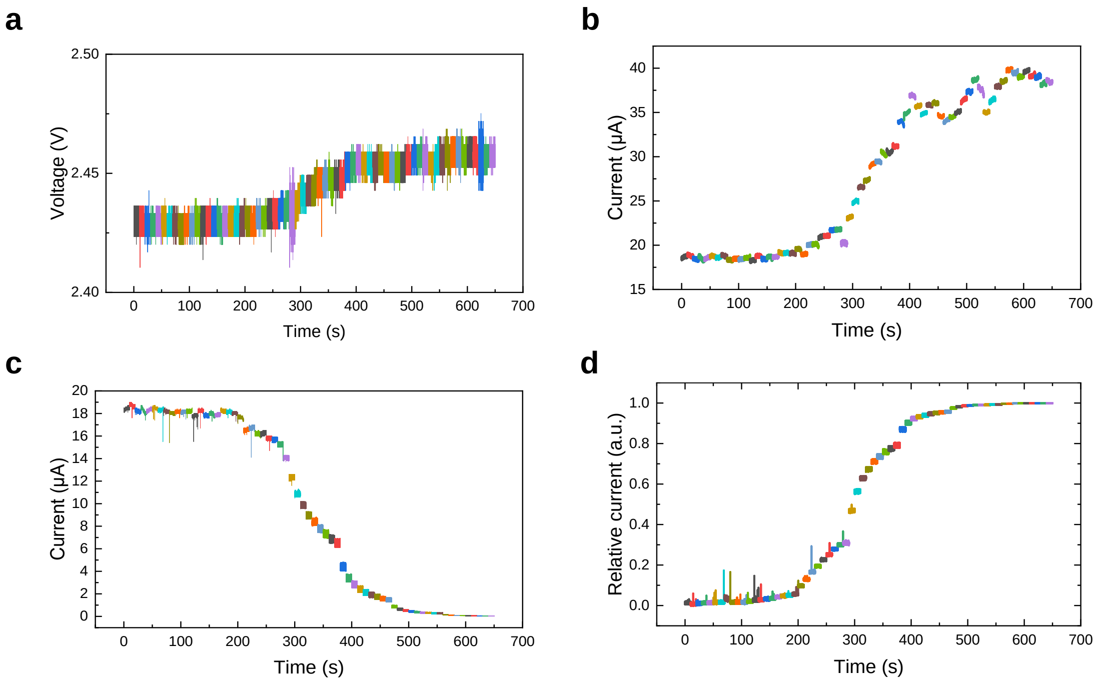

Figure S7. Electrical signals of the SPVP at different load resistance from 1 Ω to 10^7 Ω. **a** Voltage signal. Current signals of **b** the reference LED and **c** the working LED. **d** The relative current.

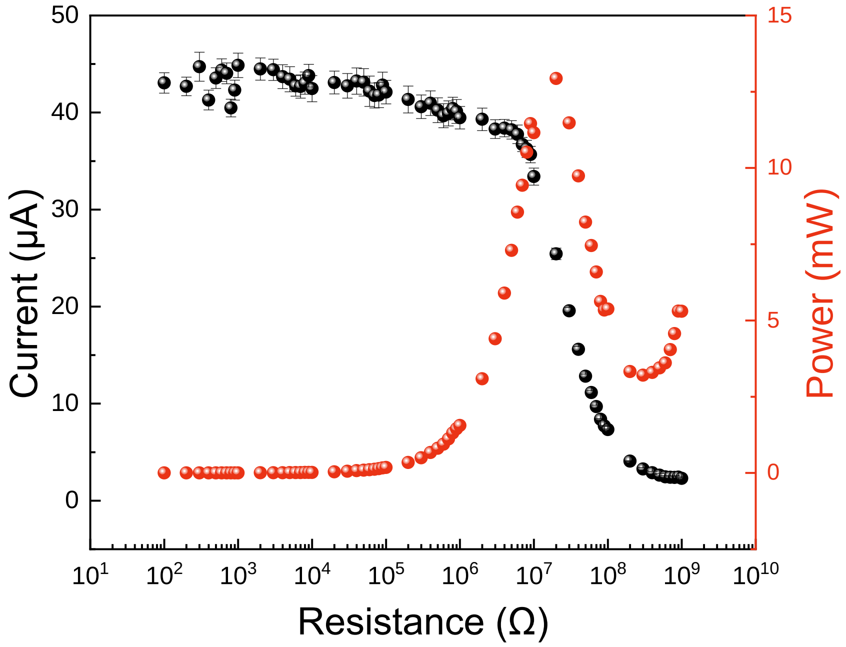

Figure S8. The current and power output of R-TENG at different loads.

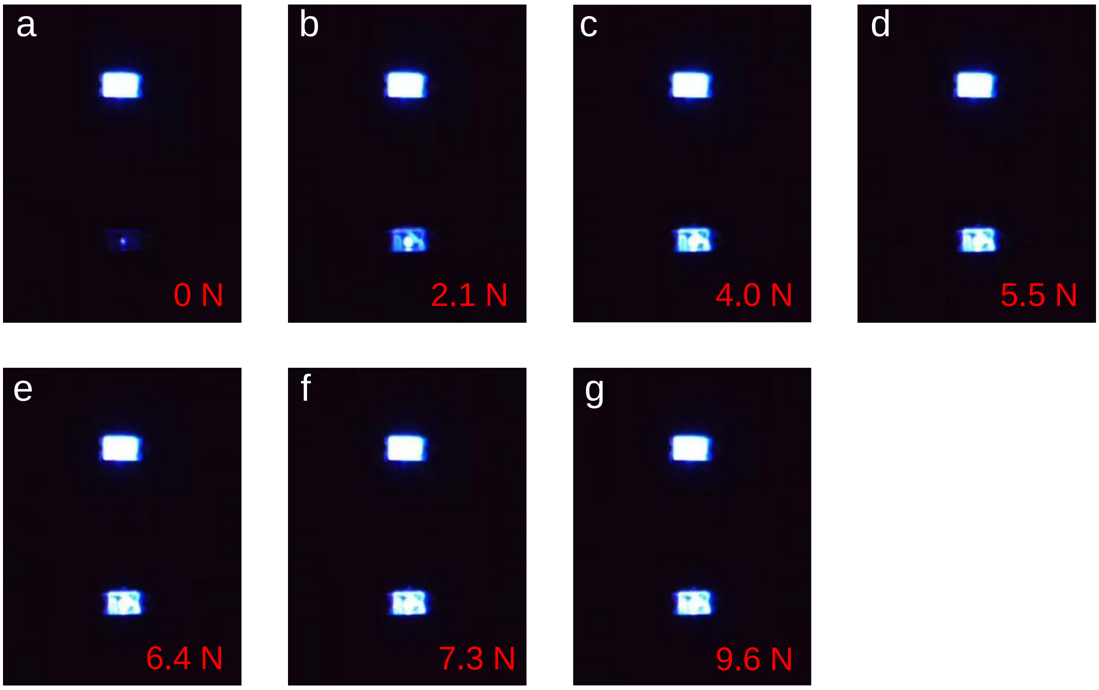

Figure S9. Light intensity of the pressure sensor with 1 kg working range at different forces. a 0 N, **b** 2.1 N, **c** 4.0 N, **d** 5.5 N, **e** 6.4 N, **f** 7.3 N, **g** 9.6 N.

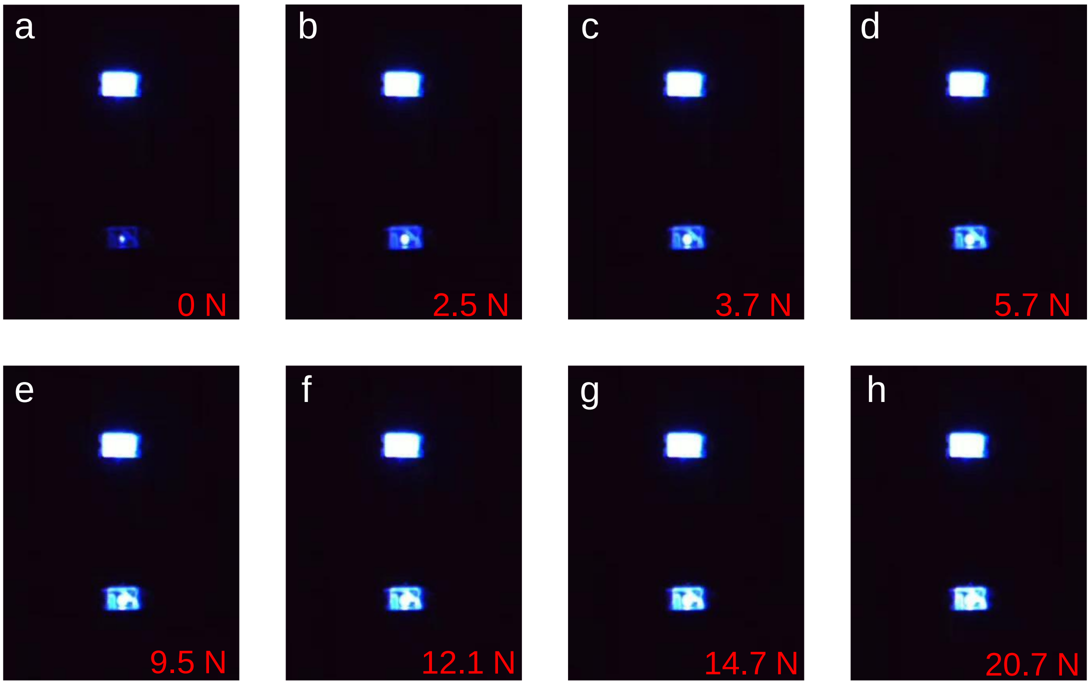

Figure S10. Light intensity change of the pressure sensor with 2 kg working range at different forces. a 0 N, **b** 2.5 N, **c** 3.7 N, **d** 5.7 N, **e** 9.5 N, **f** 12.1 N, **g** 14.7 N, **h** 20.7 N.

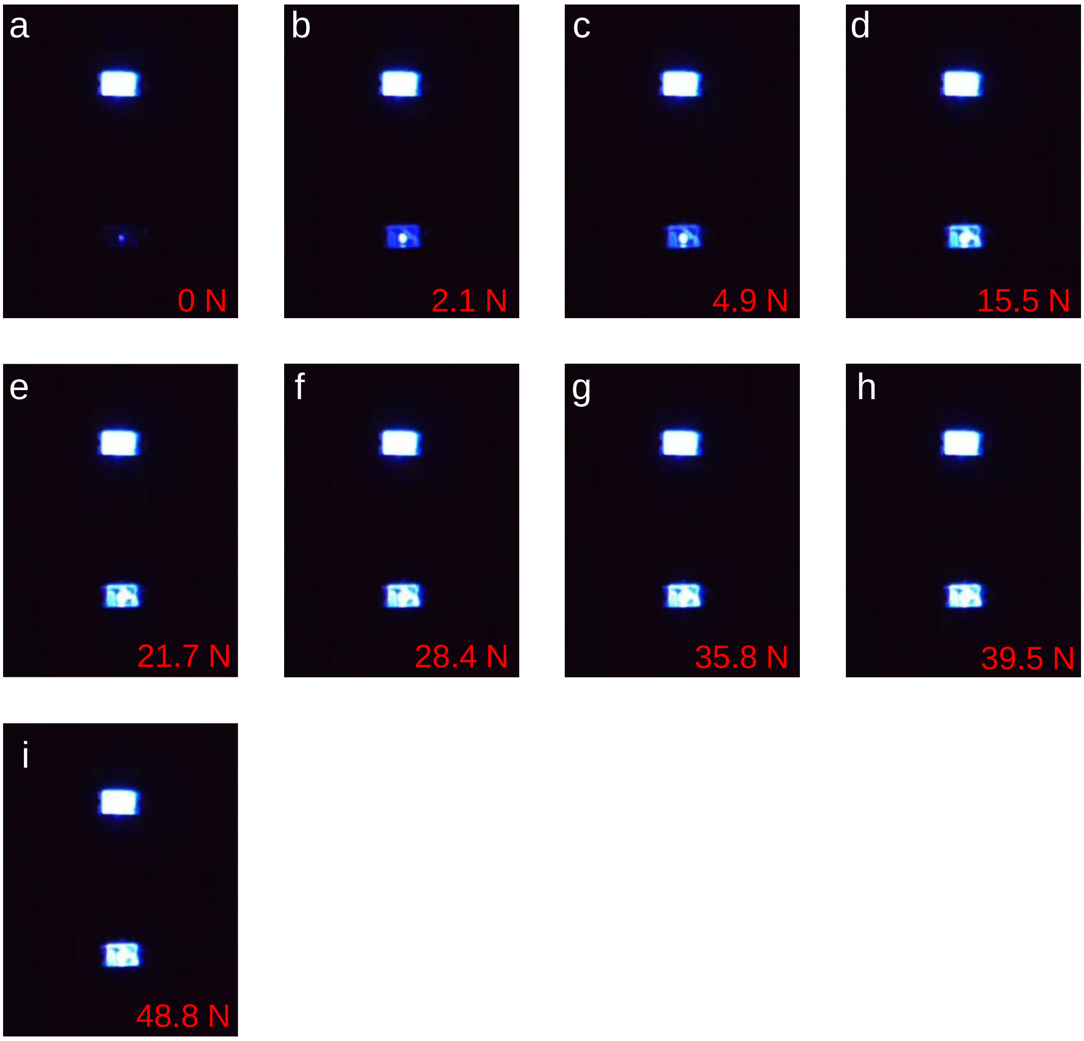

Figure S11. Light intensity change of the pressure sensor with 5 kg working range at different forces. **a** 0 N, **b** 2.1 N, **c** 4.9 N, **d** 15.5 N, **e** 21.7 N, **f** 28.4 N, **g** 35.8 N, **h** 39.5N, **i** 48.8 N.

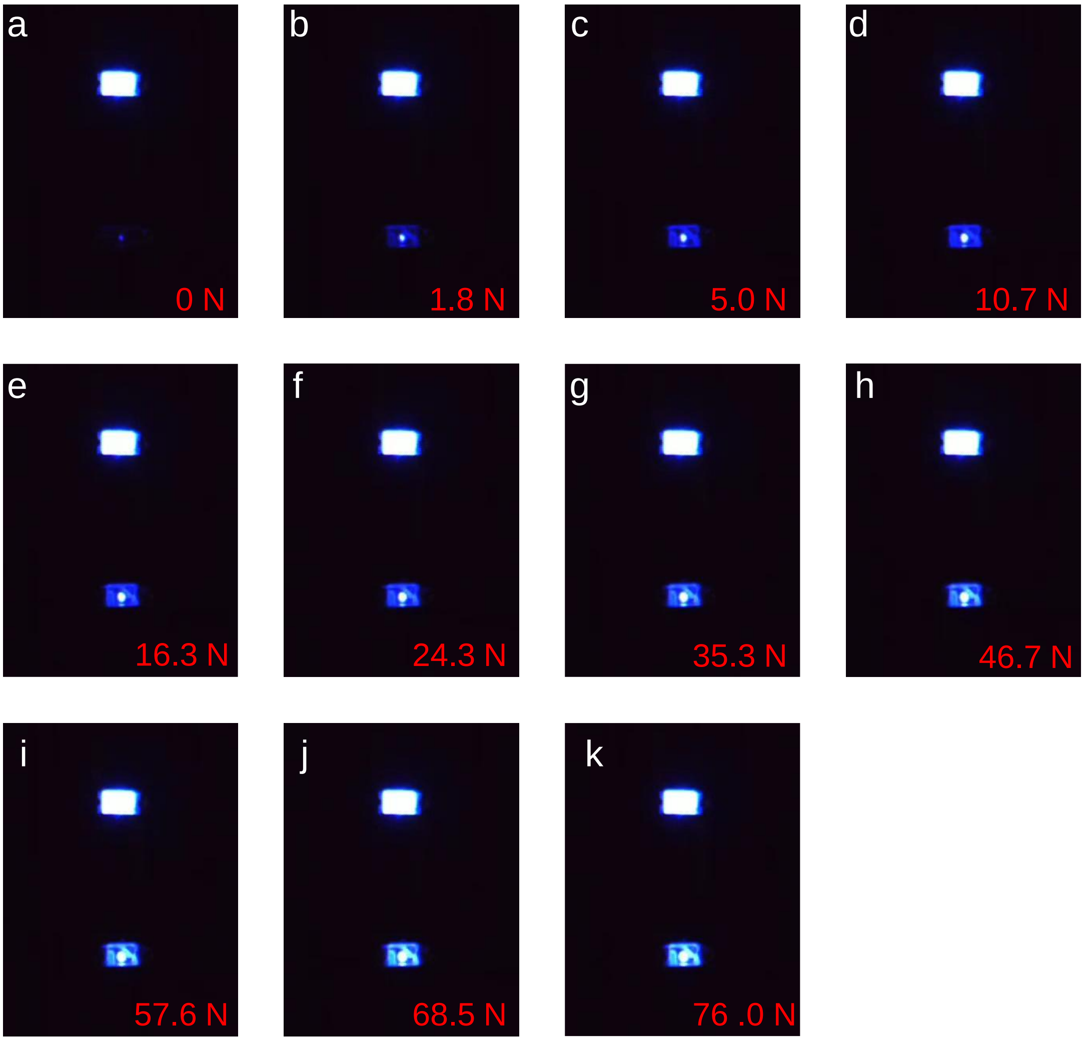

Figure S12. Light intensity change of the pressure sensor with 10 kg working range at different forces. a 0 N, **b** 1.8 N, **c** 5.0 N, **d** 10.7 N, **e** 16.3 N, **f** 24.3N, **g** 35.3 N, **h** 46.7N, **i** 57.6 N, **j** 68.5, **k** 76.0 N.

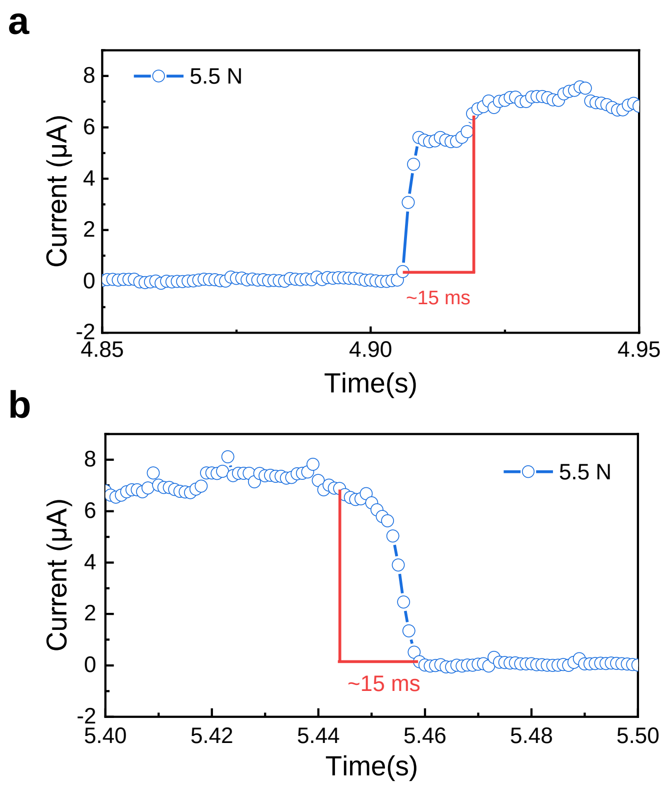

Figure S13. a Response time and **b** recovery time of the sensor (1 kg range) at 5.5 N.

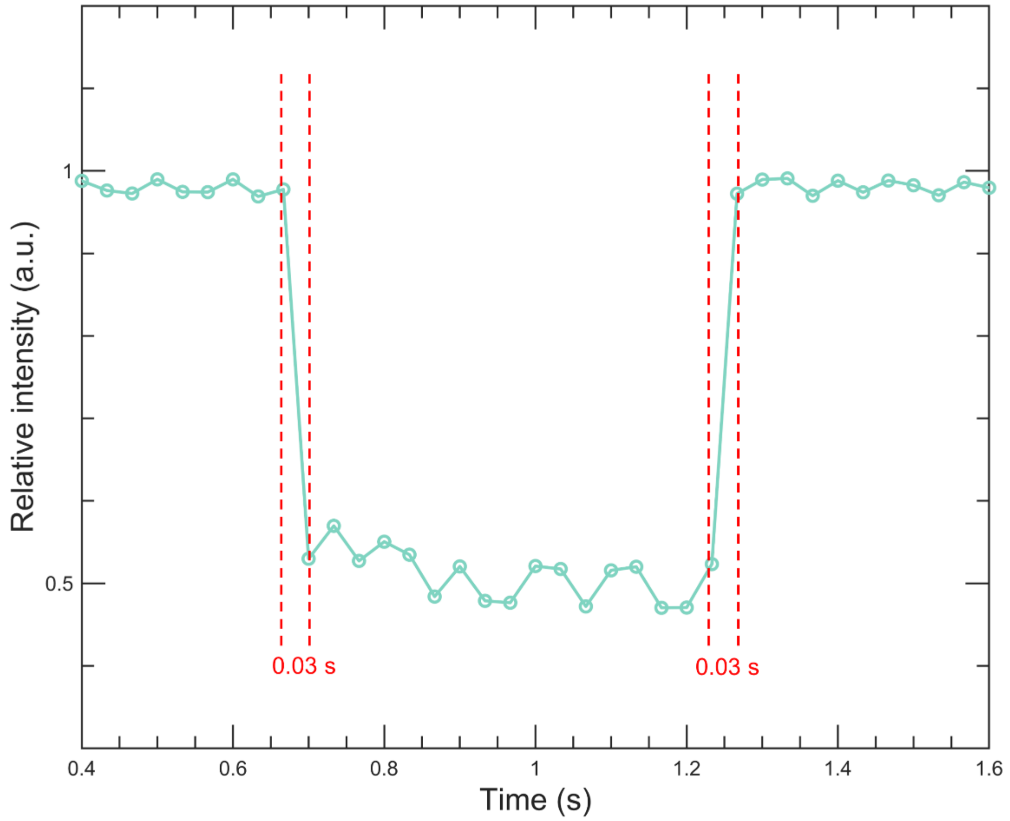

Figure S14 Optical response time and recovery time of the SPVP system.

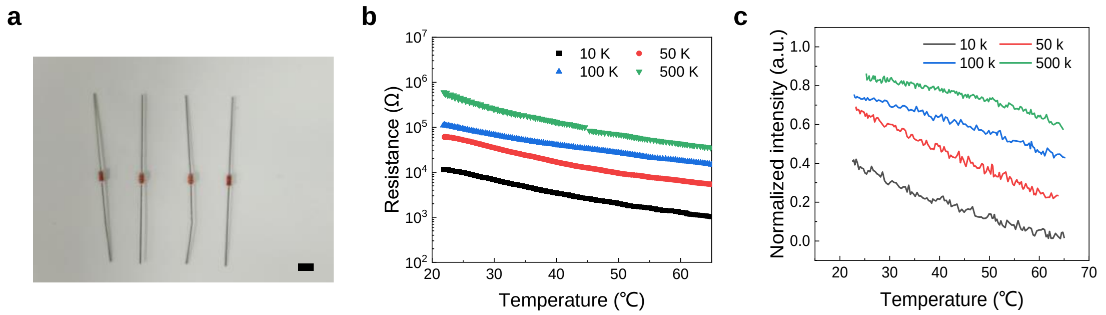

Figure S15. Electrical response and optical response of thermistors. a Photograph of thermistors (scale bar, 5 mm), **b** their resistance changes and **c** relative light intensity. Their initial resistances at room temperature are 10 kΩ, 50 kΩ, 100 kΩ, and 500 kΩ.

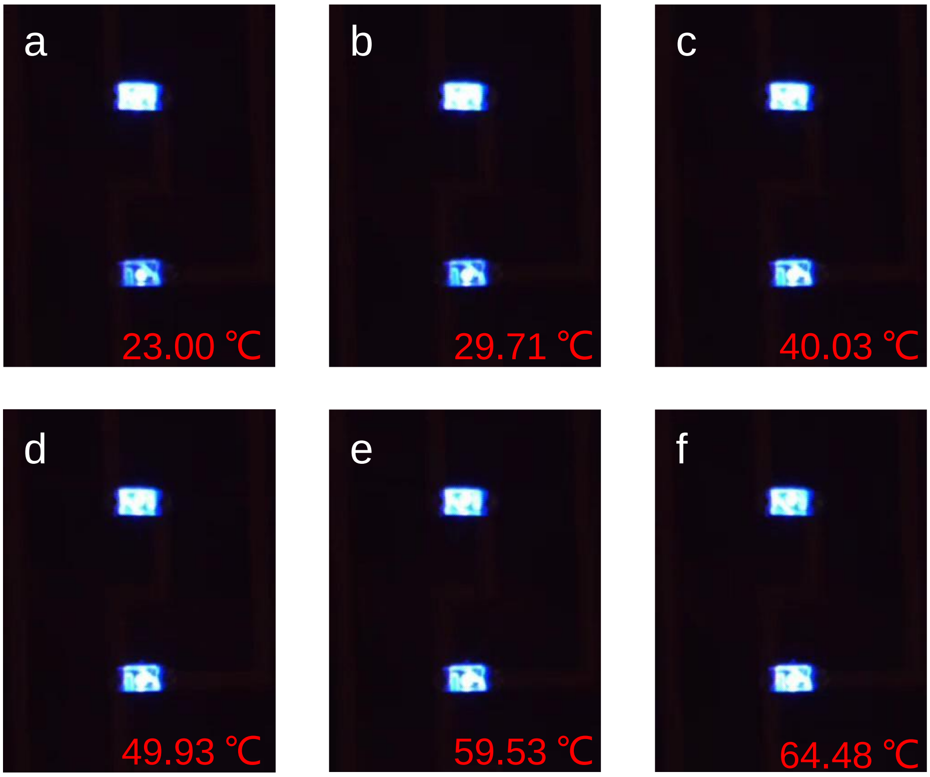

Figure S16. Light intensity change of the 10 kΩ thermistor. a 23.00 ℃, **b** 29.71 ℃, **c** 40.03 ℃, **d** 49.93 ℃, **e** 59.53 ℃, **f** 64.48 ℃.

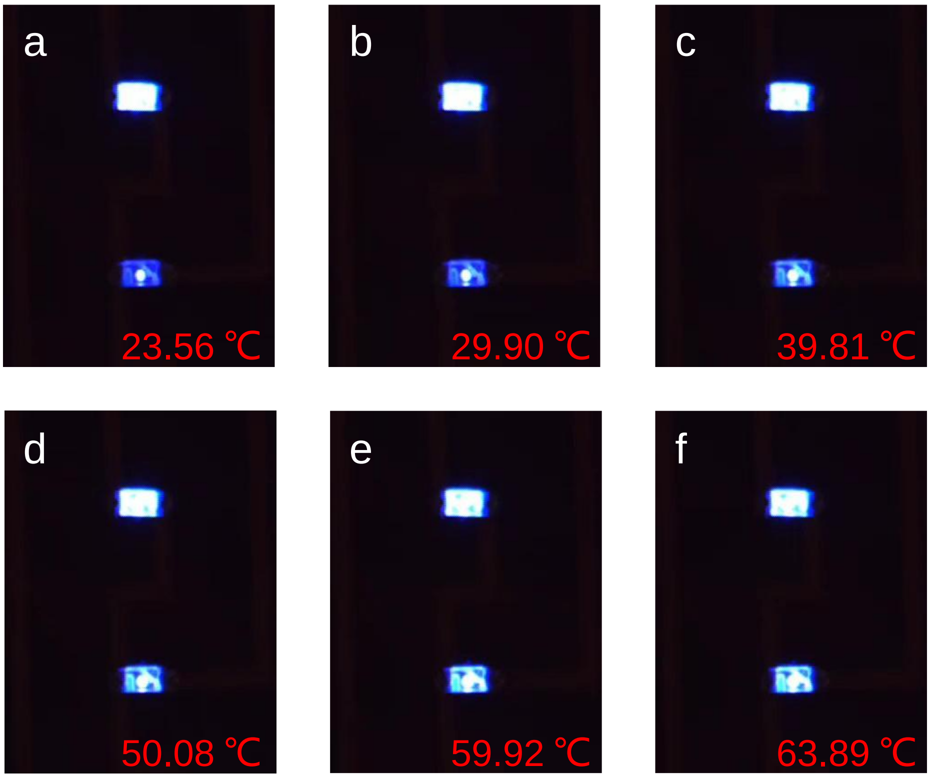

Figure S17. Light intensity change of the 50 kΩ thermistor. a 23.56 ℃, **b** 29.90 ℃, **c** 39.81 ℃, **d** 50.08 ℃, **e** 59.92 ℃, **f** 63.89 ℃.

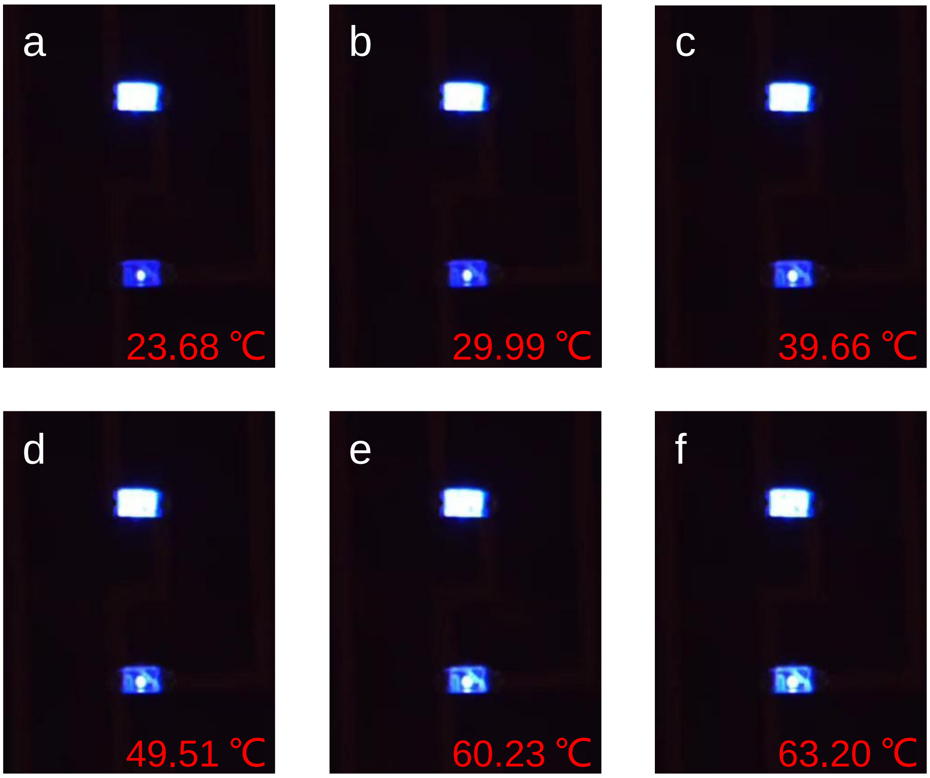

Figure S18. Light intensity change of the 100 kΩ thermistor. a 23.68 °C, **b** 29.99 °C, **c** 39.66 °C, **d** 49.51 °C, **e** 60.23 °C, **f** 63.20 °C.

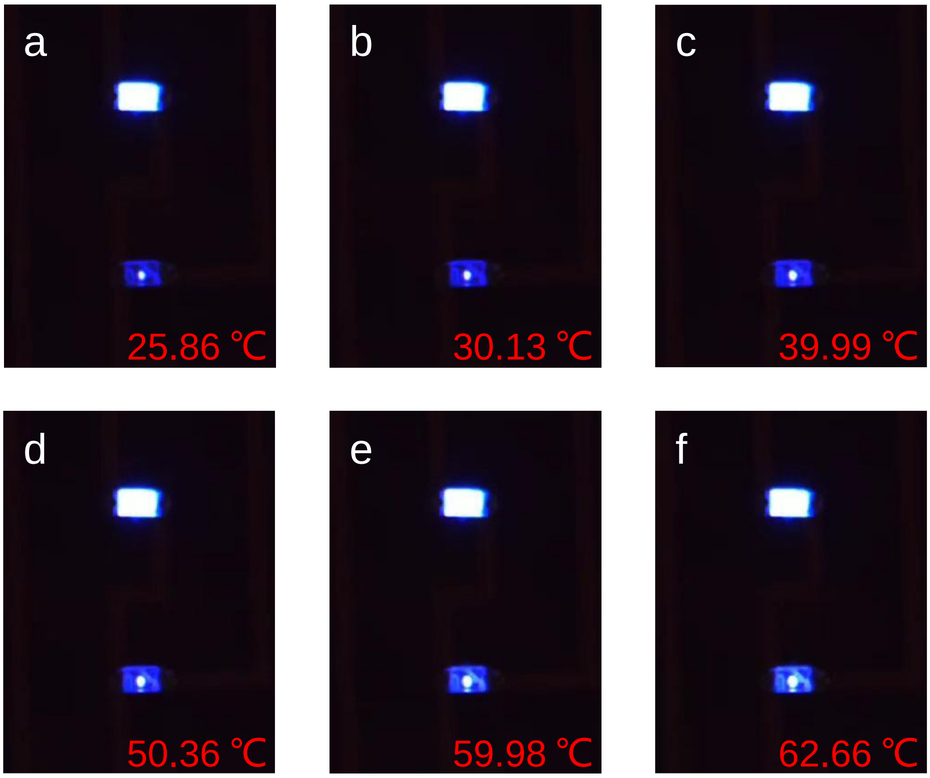

Figure S19. Light intensity change of the 10 kΩ thermistor. a 25.86 ℃, **b** 30.13 ℃, **c** 39.99 ℃, **d** 50.36 ℃, **e** 59.98 ℃, **f** 62.66 ℃.

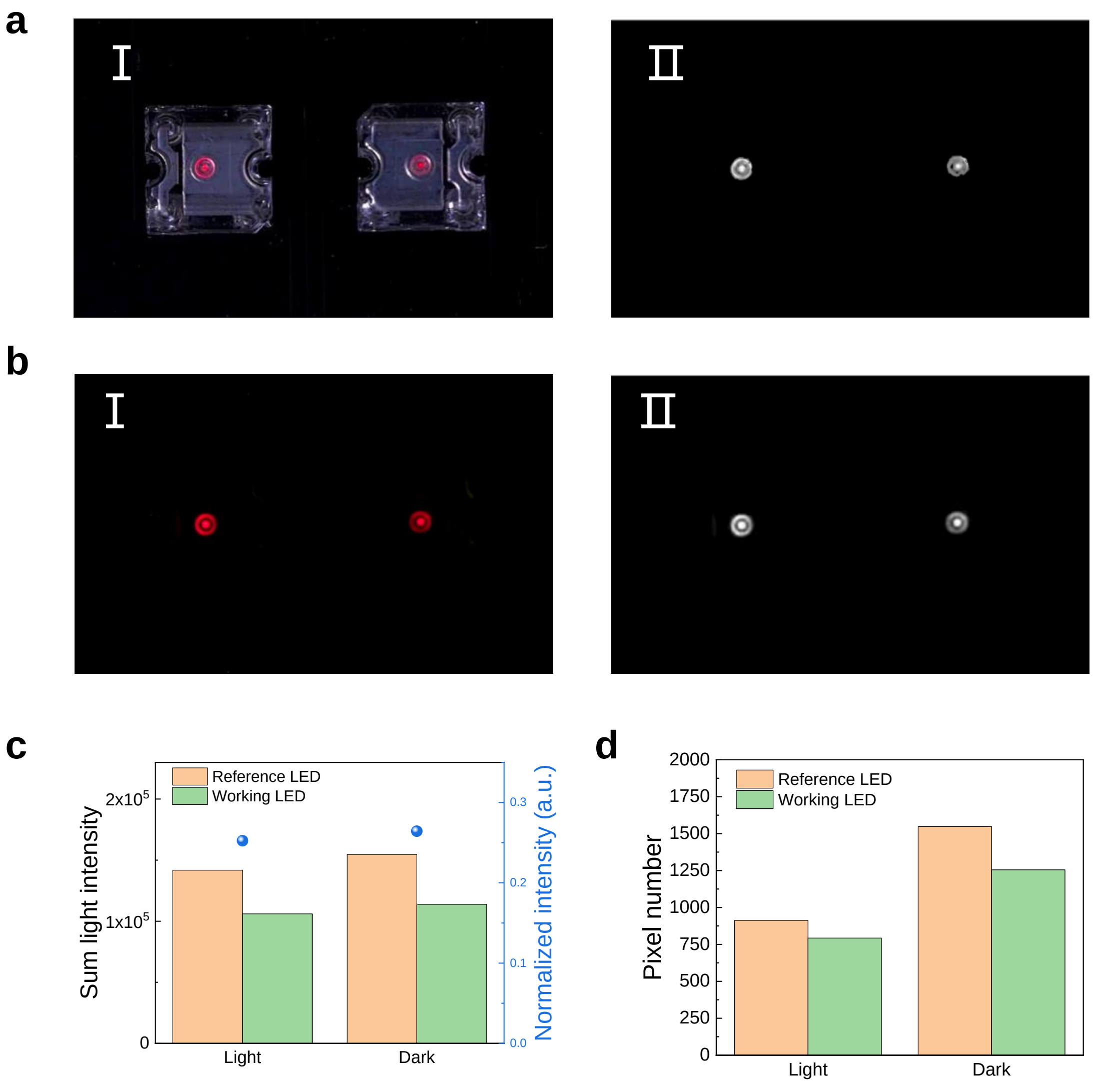

Figure S20. Comparison of the light intensity under the light and dark condition. **a** The image and extracted red component under light condition. A black background is used to increase contrast. **b** The image and extracted red component under dark condition. **c** The sum of light intensity under two conditions. **d** The red pixel number under two conditions.

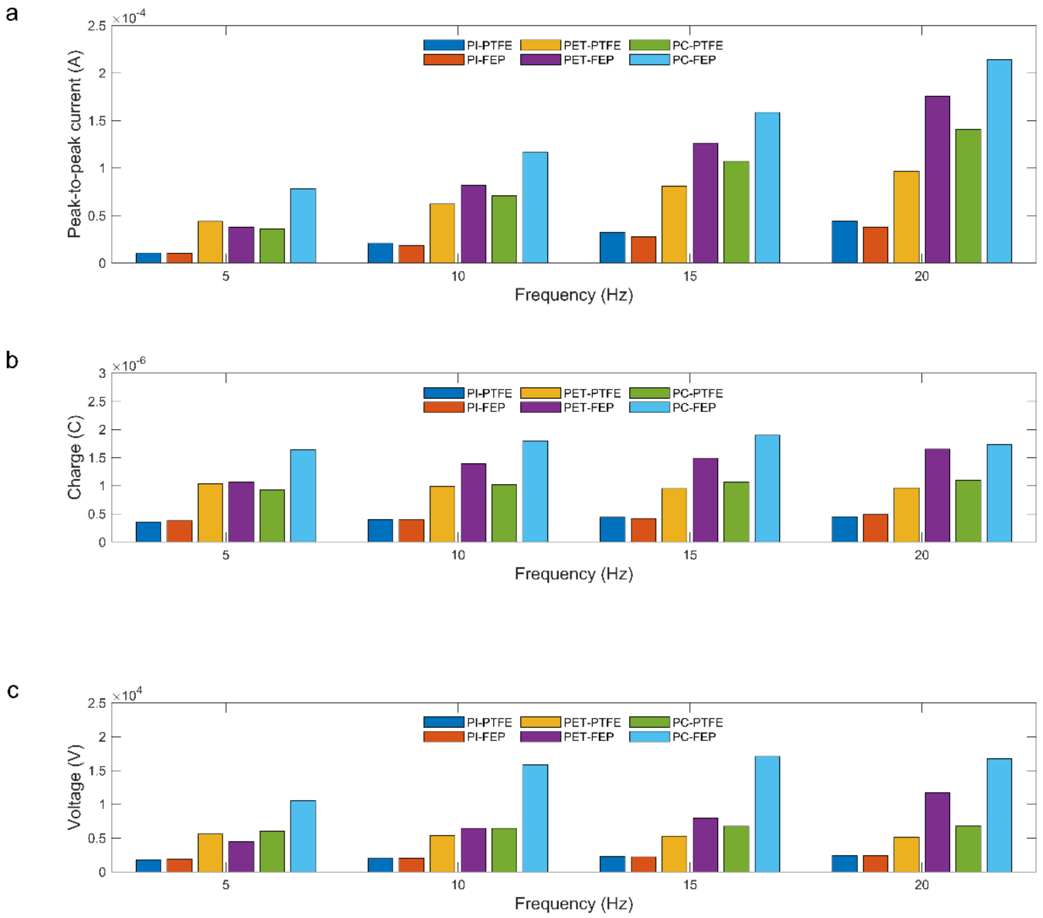

Figure S21 Output performance of different material combinations. **a** Peak-to-peak current. **b** Charge. **c** Open-circuit voltage.

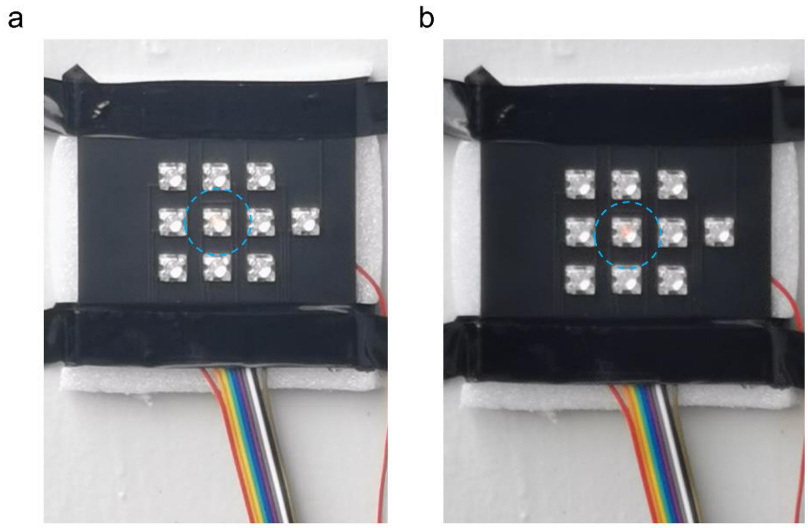

Figure S22 Comparison of the light intensity of LEDs. LEDs are driven by different material combinations: **a** PET-PTFE R-TENG and **b** FEP-PC R-TENG. The images are captured 2 m away. The light intensity of the environment is 2000 Lux.

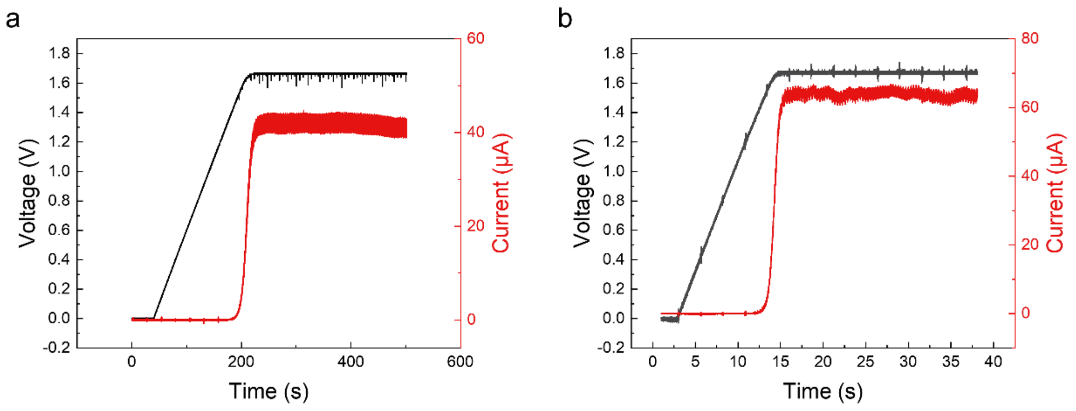

Figure S23 The voltage and current of the red LED driven by R-TENG with different materials combinations. **a** PET and PTFE. **b** PC and FEP.

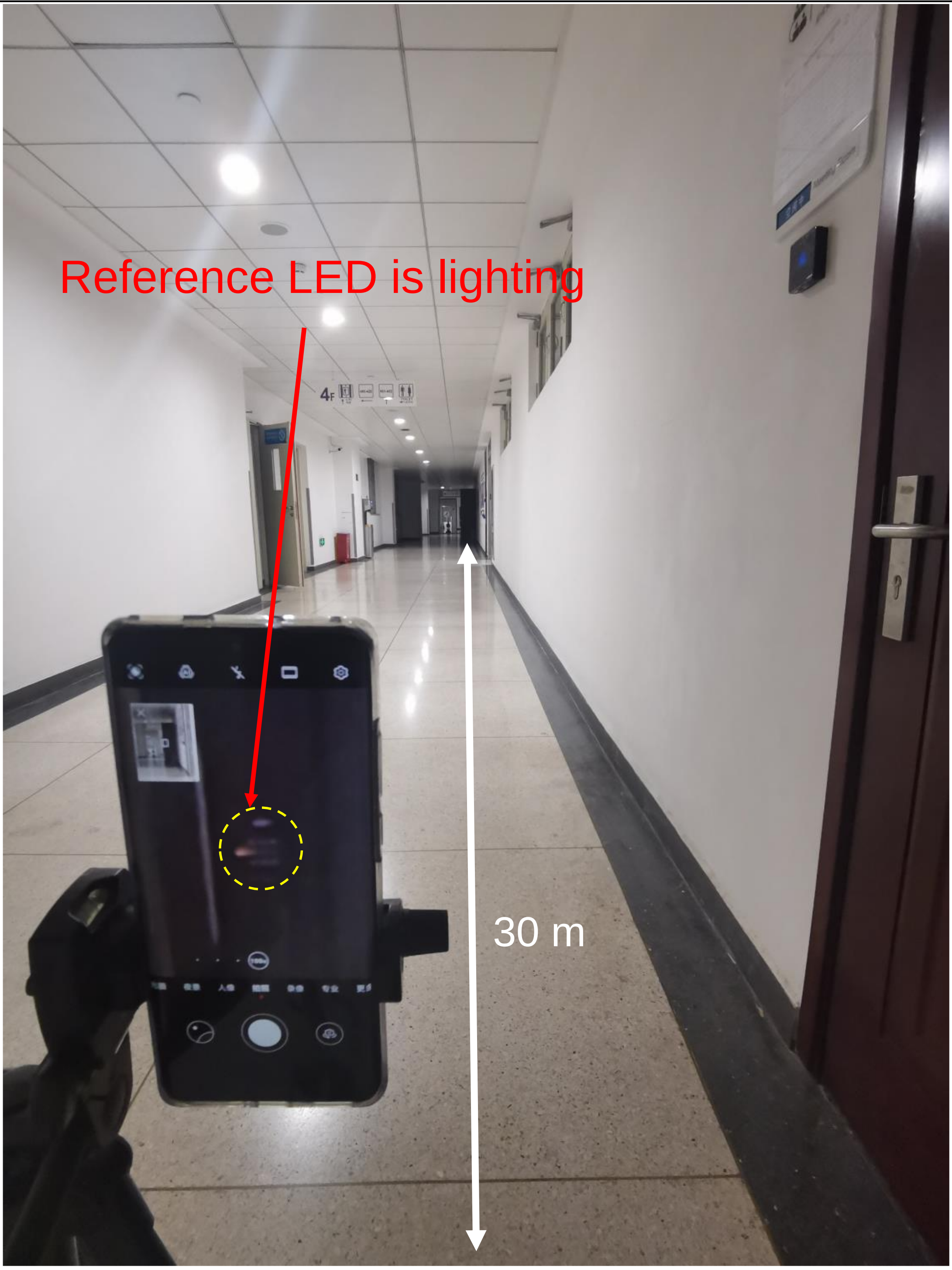

Figure S24. Transmitting the morse code over 30 m.

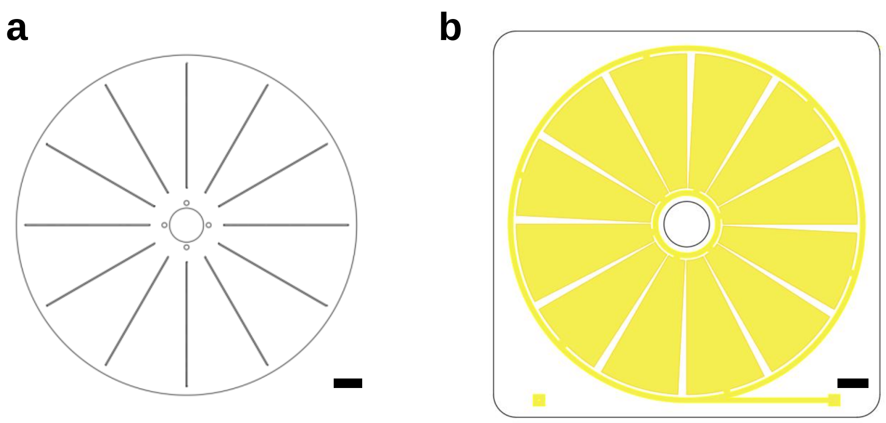

Figure S25. Shape information of the R-TENG. a rotator. **b** stator. Scale bar, 25 mm.

Supplementary Note 1

In the circuit, the capacitor can provide a relative stable output voltage and current, and thus assuming that the stable voltage V_S and current I_S would not change with the load resistance. In the whole circuit, there is:

$$\begin{cases} V_S = V_{LED} + V_R \\ I_S = I_{\mathrm{Ref}} + I_W \end{cases} \quad (1)$$

V_{LED} is the voltage of the working LED, V_R is the voltage of resistance R, I_{Ref} is the current of the reference LED, I_W is the current of the working LED.

For the working LED, the I-V curve was measured and fitted, which is shown in the Figure S26a. The relationship between I_W and V_{LED} can be described as:

$$V_{LED} = A * \left| I_W - I_0 \right|^P \quad (2)$$

Where

$$\begin{cases} A = 2.90075 \\ I_0 = 7.85754E\text{-}9 \\ P = 0.01616 \end{cases} \quad (3)$$

Also, the voltage V_R of the load resistance is:

$$V_R = I_W R \quad (4)$$

Combining Eq.(1), (2), (4), we get the relationship between I_W, I_R, R, and relative current.

$$\begin{cases} R = \dfrac{V_S - A * \left| I_W - I_0 \right|^P}{I_W} \\ I_{\mathrm{Ref}} = I_S - I_W \\ \Delta I = \dfrac{I - 2I_W}{I - I_W} \end{cases} \quad (5)$$

For the simulation, parameters are:

$$\begin{cases} V_S = 2.432\ V \\ I_S = 3.74E\text{-}5\ \mathrm{A} \\ I_W = [20E-9, 1.85E-5]\ \mathrm{A} \end{cases} \quad (5)$$

in which, V_S, I_S, I_W were the measured values at initial condition. In the Fig. 2c-e, the simulation results and measured data are fitted well.

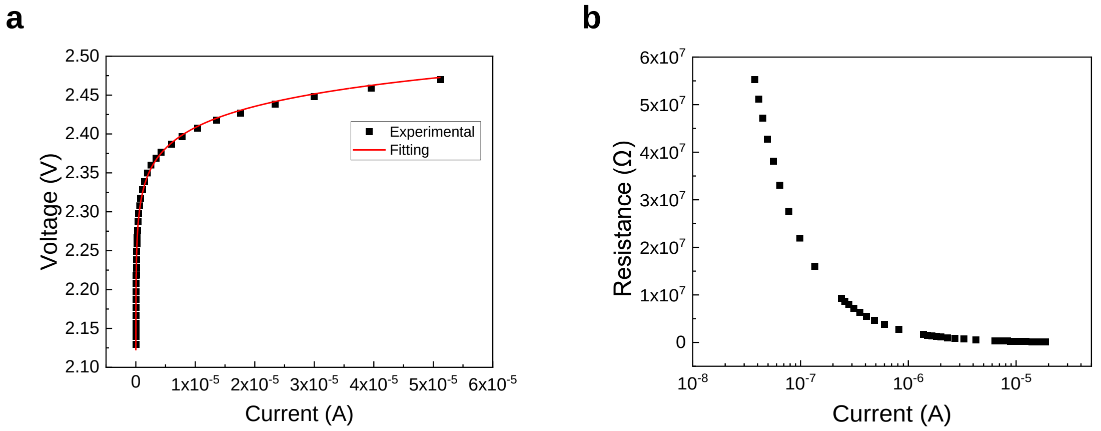

Figure S26. a I-V curve of the blue LED, and corresponding **b** resistance change.
